# Highly Specific Sigma Receptor Ligands Exhibit Anti-Viral Properties in SARS-CoV-2 Infected Cells

**DOI:** 10.3390/pathogens10111514

**Published:** 2021-11-20

**Authors:** David A. Ostrov, Andrew P. Bluhm, Danmeng Li, Juveriya Qamar Khan, Megha Rohamare, Karthic Rajamanickam, Kalpana K. Bhanumathy, Jocelyne Lew, Darryl Falzarano, Franco J. Vizeacoumar, Joyce A. Wilson, Marco Mottinelli, Siva Rama Raju Kanumuri, Abhisheak Sharma, Christopher R. McCurdy, Michael H. Norris

**Affiliations:** 1 Department of Pathology, Immunology and Laboratory Medicine, University of Florida College of Medicine, Gainesville, FL 32610, USA; ostroda@pathology.ufl.edu (D.A.O.); dameng1986@ufl.edu (D.L.); 2 Department of Geography, University of Florida College of Liberal Arts and Sciences, Gainesville, FL 32611, USA; abluhm@ufl.edu; 3 Emerging Pathogens Institute, University of Florida, Gainesville, FL 32610, USA; 4 Department of Biochemistry, Microbiology, and Immunology, University of Saskatchewan, Saskatoon, SK S7N 5E5, Canada; jqk913@mail.usask.ca (J.Q.K.); mrr968@mail.usask.ca (M.R.); joyce.wilson@usask.ca (J.A.W.); 5 Division of Oncology, College of Medicine, University of Saskatchewan, Saskatoon, SK S7N 5E5, Canada; kar029@mail.usask.ca (K.R.); kak677@mail.usask.ca (K.K.B.); franco.vizeacoumar@usask.ca (F.J.V.); 6 Vaccine and Infectious Disease Organization-International Vaccine Centre, University of Saskatchewan, Saskatoon, SK S7N 5E3, Canada; jocelyne.lew@usask.ca (J.L.); darryl.falzarano@usask.ca (D.F.); 7 Cancer Research Department, Saskatchewan Cancer Agency, Saskatoon, SK S7N 5E5, Canada; 8 Department of Medicinal Chemistry, University of Florida College of Pharmacy, Gainesville, FL 32610, USA; m.mottinelli@cop.ufl.edu (M.M.); cmccurdy@cop.ufl.edu (C.R.M.); 9 Department of Pharmaceutics, University of Florida College of Pharmacy, Gainesville, FL 32610, USA; kanumuris@cop.ufl.edu (S.R.R.K.); asharma1@cop.ufl.edu (A.S.); 10 Translational Drug Development Core, University of Florida Clinical and Translational Sciences Institute, Gainesville, FL 32610, USA

**Keywords:** drug discovery, sigma-1 receptor, SARS-CoV-2, molecular docking, antiviral therapeutics

## Abstract

(1) Background: There is a strong need for prevention and treatment strategies for COVID-19 that are not impacted by SARS-CoV-2 mutations emerging in variants of concern. After virus infection, host ER resident sigma receptors form direct interactions with non-structural SARS-CoV-2 proteins present in the replication complex. (2) Methods: In this work, highly specific sigma receptor ligands were investigated for their ability to inhibit both SARS-CoV-2 genome replication and virus induced cellular toxicity. This study found antiviral activity associated with agonism of the sigma-1 receptor (e.g., SA4503), ligation of the sigma-2 receptor (e.g., CM398), and a combination of the two pathways (e.g., AZ66). (3) Results: Intermolecular contacts between these ligands and sigma receptors were identified by structural modeling. (4) Conclusions: Sigma receptor ligands and drugs with off-target sigma receptor binding characteristics were effective at inhibiting SARS-CoV-2 infection in primate and human cells, representing a potential therapeutic avenue for COVID-19 prevention and treatment.

## 1. Introduction

There is a strong need for safe drugs and vaccines to target emerging pathogens such as SARS-CoV-2. Although recent studies identified approved drugs that exhibit antiviral activities against SARS-CoV-2 [1,2], current therapeutic treatment strategies for COVID-19 have limited effectiveness. There are currently no oral medications given emergency use authorization from the Food and Drug Administration to prevent SARS-CoV-2 infection or to treat COVID-19. There is an urgent need to identify safe, economical, orally deliverable approved drugs with activity against SARS-CoV-2 to prevent infection in at-risk populations, and to treat patients experiencing viral disease [3]. Attempts to identify approved drugs with antiviral activity led to the discovery of more than 100 compounds that exhibit direct antiviral activity against SARS-CoV-2 isolates in vitro [2,4,5,6]. Although the on- and off-target binding mechanisms that mediate anti-SARS-CoV-2 activity are not clear, two classes of molecules were previously found to effectively inhibit virus infectivity: protein biogenesis inhibitors (e.g., zotatifin, ternatin-4, PS3061) and ligands of the sigma-1 and sigma-2 receptors (e.g., haloperidol, clemastine, cloperastine) [7].

Specific antihistamines with off-target antiviral activity may have repurposed utility for prevention and treatment of COVID-19 because of known safety profiles and widespread availability. Common antihistamines that exhibit off-target antiviral activity include hydroxyzine, azelastine and diphenhydramine [8]. Mechanisms of action for drugs with direct anti-SARS-CoV-2 activity have important clinical implications in terms of dosing and drug interactions. Defining mechanisms that drive antiviral activity against SARS-CoV-2 will provide rationale for drug combinations targeting distinct antiviral pathways [9]. Drug combinations that target separate antiviral pathways are expected to inhibit drug resistant variants resulting from emerging mutations.

Coronaviruses replicate in a modified compartment derived from the endoplasmic reticulum (ER). The sigma receptor-1 is an ER resident chaperone that normally functions to modulate the ER stress response [10]. Coronavirus infection activates pathways to facilitate adaptation of ER stress to virus proliferation. These pathways are thought to hijack the host cell ER stress response to modulate protein translation, ER protein folding capacity and ER-associated degradation. Targeting the ER stress response could elucidate coronavirus protein-host interactions and provide rationale for new therapeutic approaches to prevention and treatment of COVID-19.

In infected cells, the sigma-1 receptor was shown to link the SARS-CoV-2 replicase/transcriptase complex to the ER membrane by binding directly to nonstructural protein 6 (NSP6) [7]. Although sigma-1 receptor ligands exert antiviral activity against non-coronaviruses and coronaviruses [10], it is not known if agonist or antagonist activities prevent SARS-CoV-2 infection. Understanding binding interactions of antiviral sigma-1 receptor ligands may provide the basis for drug development and optimization. 

Although approved drugs that inhibit SARS-CoV-2 in vitro have been shown to bind sigma-2 receptors, the structure and functions of sigma-2 receptors are not well characterized or understood [11]. Sigma-1 and sigma-2 receptors are unrelated in sequence and structure. The sigma-2 receptor is an ER resident membrane protein thought to be involved in hormone, calcium and neuronal signaling [12]. The sigma-2 receptor regulates cholesterol transport and contributes to cholesterol homeostasis [13]. In infected cells, the sigma-2 receptor was shown to bind directly to SARS-CoV-2 ORF9c [7], suggesting that sigma-2 receptor ligands may block host protein:virus protein interactions. Recently, the sigma-1 and -2 ligand PB28 that had sub-nanomolar in vitro SARS-CoV-2 inhibitory effects was found ineffective in vivo (33). The hypothesized cause for poor efficacy was that this compound induced high levels of phospholipidosis in vitro that resulted in virus inhibition that could not be achieved in vivo. Sigma receptor ligands, as a class, should not be discounted because of the poor performance of a single compound. In this work, we demonstrate phospholipidosis induction by sigma ligands does not correlate with inhibition of SARS-CoV-2 viral replication.

**Figure 1:**
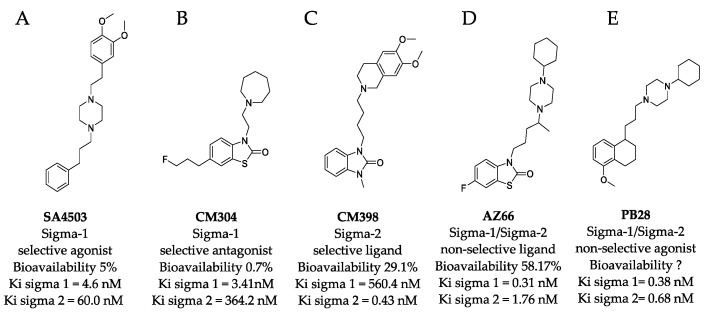
**Structures of sigma ligands utilized in this and other studies.** The structure of sigma ligands important to this study are presented with the sigma specific activity, Ki for the respective sigma receptor binding property, and the bioavailability if known. (**A**) SA4503 (cutamesine) is a selective Sigma-1 piperazine agonist with > 15-fold preference for sigma-1 over sigma-2. (**B**) CM304 is a highly selective benzothiazolone sigma-1 antagonist. (**C**) CM398 is a highly selective benzimidazolone-based sigma-2 ligand. (**D**) AZ66 is a mixed sigma-1/sigma-2 selective ligand with an optimized pharmacokinetic profile. (**E**) PB28 is not utilized in this study but recent work demonstrated its utility in vitro was compromised by its toxicity and is included as a structural comparison to the other compounds tested in this work.

It is clear that multiple sigma receptor ligands exhibit antiviral properties against SARS-CoV-2, but the relative roles of the sigma-1 receptor and sigma-2 receptor agonism and antagonism in modulating antiviral activities are not known. In this study, antiviral activities of highly selective ligands (Figure 1) were measured to define mechanisms driving inhibition of SARS-CoV-2 infection in vitro: a sigma-1 receptor specific agonist (SA4503, cutamesine) [14,15,16], sigma-1 receptor antagonist (CM304) [17], sigma-2 receptor specific ligand (CM398) [18], and a mixed affinity sigma-1/sigma-2 ligand (AZ66) [19,20]. The benzothiazolone (CM304 and AZ66) and benzimidazolone (CM398) containing compounds were selected for their demonstrated selectivity for sigma receptors against other aminergic transporters or receptors [21]. In addition, the specific compounds were chosen for their differential affinity at the two receptor subtypes, aiming to clarify the involvement of each receptor in the inhibition of SARS-CoV-2 infection in vitro.

## 2. Results

### 2.1. Sigma Ligands Inhibit SARS-CoV-2-Mediated Cell Death, Intracellular Replication, and Infectivity 

**Figure 2:**
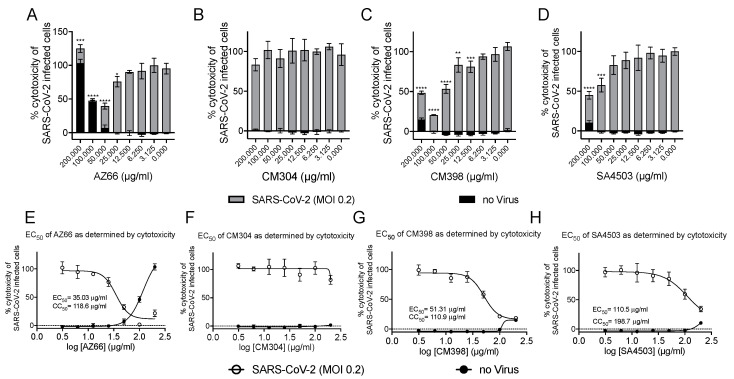
**Highly specific sigma ligands inhibit SARS-CoV-2-induced cytotoxicity in Vero E6 cells.** Cytotoxicity measured by LDH release in Vero E6 cells at 72 h in the presence of the indicated drug concentration alone (black bars) or in the presence of the indicated drug after infection with SARS-CoV-2 at an MOI of 0.2 (gray bars) (**A**–**D**). The 50% cytotoxic concentration (CC_50_) of the drug alone (black circles) and the 50% effective concentration (EC_50_) at which the drug inhibits SARS-CoV-2-induced cytotoxicity (black squares) as determined by non-linear regression for each drug is shown in (**E**–**H**). The calculated CC_50_ and EC_50_ are shown when appropriate. Data points were obtained from triplicate experiments. Similar results were obtained from an infection with MOI of 0.3 however the dynamic range was not as satisfactory. *, *p* ≤ 0.05; **, *p* ≤ 0.01; ***, *p* ≤ 0.001; ****, *p* ≤ 0.0001.

We utilized several assays to determine antiviral efficacy of sigma ligands. The African green monkey cell line Vero E6 has been shown to support SARS-CoV-2 infections and was the primary cell line used in our in vitro assays. Initial experiments measuring reduction of SARS-CoV-2 mediated cytotoxicity were carried out at MOI 0.1 and indicated ligands that specifically and non-specifically target sigma receptors had potential for further analysis. With ligand concentrations used in these preliminary studies as a starting point, toxicity and antiviral activities were measured in cytotoxicity assays. In Figure 2A–D, toxicity of ligands alone is observed by the black bars. Superimposed on the black bars are the cytotoxicity values (gray bars) of cells in the presence of the indicated ligand concentrations. The cytotoxic concentration of ligand alone (CC_50_) and effective concentrations (EC_50_) of each sigma ligand for inhibition of SARS-CoV-2 induced cytotoxicity were determined by non-linear regression (Figure 2E–H). The specific sigma-1 and sigma-2 receptor ligand AZ66 had the lowest EC_50_ of the ligands tested at 86.4 μM as measured by cytotoxicity. The sigma-1 receptor antagonist CM304 showed insignificant viral inhibition. However, the sigma-1 receptor agonist SA4503 (cutamesine) had moderate inhibitory activity against SARS-CoV-2-induced cytotoxicity at 299.9 μM. The highly specific sigma 2- receptor ligand CM398 showed significant inhibitory activity and had an EC_50_ of 129.7 μM. Of the ligands tested in this work, AZ66 has the highest selectivity index (SI; CC_50_/EC_50_) ratio as measured by cytotoxicity (>2.82). To compare AZ66 to previously published studies done with remdesivir, a plaque assay was utilized to measure EC_50._ The reported SI for remdesivir was >4.96 [6] while AZ66 was >19.72 by plaque assay in this work, meaning the gap between SARS-CoV-2 inhibitory concentrations and toxic concentrations is greater for AZ66 than remdesivir (Table 1). The differences in compound efficacy are linked to the ligand binding strength (the lower the Ki) and activity at the sigma receptors (agonism vs. antagonism). The higher EC_50_ levels from the cytotoxicity assay versus the plaque assay indicate the more sensitive nature of measuring infectious particles. Even low levels of virus can cause cell damage thus requiring higher levels of drug to completely eradicate virus-mediated cytotoxicity.

**Figure 3:**
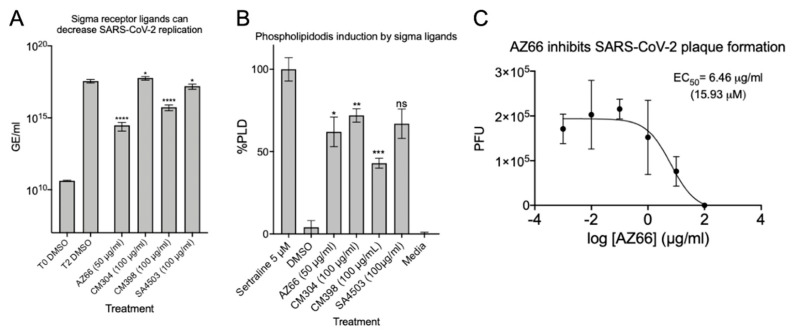
Sigma ligands AZ66, CM398, and SA4503 significantly reduce viral genome replication and AZ66 potently reduces SARS-CoV-2 plaque formation. (**A**), SARS-CoV-2 was used to infect Vero E6 monolayers at an MOI of 0.01 in the presence of 50 μg/mL AZ66, 100 μg/mL for each of CM304, CM398 or SA4503, or 1% DMSO in triplicate. After 48 h, cell monolayers were harvested into AVL buffer and RNA was isolated with the QIAamp viral RNA Kit and qPCR was used to enumerate N copies per ml and are presented as genomic equivalents (GE) per ml. The T0 DMSO treatment represents the input GE/mL harvested immediately after virus addition to the monolayers. The data is the mean and standard deviation of three experiments. (**B**), Phospholipidosis in H23 cells was measured after 48 h of treatment with the indicated sigma ligand concentration. One-way ANOVA indicates significant difference compared to the sertraline positive control. (**C**), Plaque reduction assay showed the sigma ligand AZ66 was highly effective at inhibiting plaque formation by the SARS-CoV-2 virus. The EC_50_ of AZ66 in this assay was 6.46 μg/mL (15.93 μM). The calculated EC_50_ of AZ66 by both cytotoxicity assay and plaque reduction assay is well below the published area under the curve (mean 158.22 μg h/mL) in rats following oral dosing of 20 mg/kg and also below the AUC following an intravenous 5 mg/kg dose (mean 63.2 μg h/mL). *, *p* ≤ 0.05; ****, *p* ≤ 0.0001.

After verifying the ability of ligands to inhibit or reduce cytopathic effects, we infected Vero E6 cells at a low MOI and quantified viral replication through qPCR. Quantitative analysis was used to determine if each ligand inhibited replication of the SARS-CoV-2 genome. The data in Figure 3A show that in the presence of 50 μg/mL AZ66 viral replication is reduced by 99.9% (3-log). At 100 μg/mL, CM398 is able to reduce viral replication by 97.5% and SA4503 by 56.2%. CM304 was unable to reduce viral replication and RNA levels indicated 61.2% more virus was detected, compared to the 48 h DMSO control. To measure whether the inhibitory activity of ligands were correlated with accumulation of phospholipids in cell membranes as a non-specific in vitro SARS-CoV-2 inhibitor, phospholipidosis was measured in H23 human lung epithelial cells (Figure 3B). CM304 showed the highest mean levels of phospholipidosis followed by SA4503, AZ66, and CM398. CM304 was the least effective SARS-CoV-2 inhibitor tested while having the highest phospholipid accumulation, suggesting a correlation of antiviral activity and phospholipidosis levels was not identified. Since the sigma-1 and -2 ligand AZ66 was the most potent SARS-CoV-2 cytotoxicity and replication inhibitor of those tested, we evaluated its ability to inhibit plaque formation caused by SARS-CoV-2 (Figure 3C). The EC_50_ of AZ66 as determined by plaque reduction assay was 6.46 μg/mL (15.93 μM). In a previous work, the area under the curve (AUC) for AZ66 was 158.22 μg·h/mL following a 20 mg/kg p.o. (oral) dose in rats [22]. The AUC is a measure of tissue exposure to a compound over a period of time. The high AUC for AZ66 indicates in vivo therapeutic levels of AZ66 could be achieved for COVID19 at concentrations that may not induce high levels of phospholipidosis in vitro.

**Figure 4:**
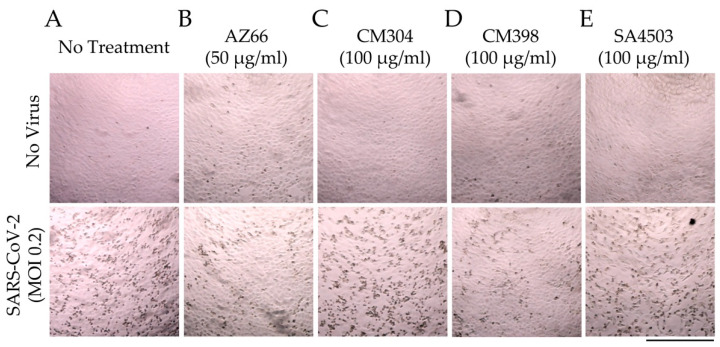
**Sigma ligands reduce SARS-CoV-2 induced cytopathic effects in cell monolayers.** Monolayers of Vero E6 cells in 96-well plates were imaged after 72 h in the absence of treatment (**A**) or in the presence of AZ66 (**B**), CM304 (**C**), CM398 (**D**), or SA4503 (**E**) alone (top panels). In the bottom panel are images of monolayers infected with SARS-CoV-2 at an MOI of 0.2 in the presence of the same drug treatments as in the top panel. Cytopathic effects (CPE) in the monolayers caused by SARS-CoV-2 infection are visible as dark puncta (dead infected cells) against the light-colored intact monolayer. These puncta are absent from the uninfected monolayer images (top panel) and are greatly reduced in number in the AZ66 and CM398-treated infected monolayers (**B**,**D**; bottom panels) compared to untreated infected monolayers (**A**, bottom panel). The scale bar at lower right is equal to 500 μm.

These quantitative data were verified by microscopic evaluation of infected monolayers (Figure 4). The ability of AZ66 and CM398 to greatly reduce cytopathic effects (CPE) caused by SARS-CoV-2 infection is evident as a decrease in cell-rounding and cell death (dark spots, Figure 4B,D) compared to the no treatment controls (Figure 4A). CM304 and SA4503 were unable to visibly reduce CPE (Figure 4C,E). Collectively, these data indicate that antiviral activity against SARS-CoV-2 was driven by agonism of the sigma-1 receptor (e.g., SA4503), and by ligation of the sigma-2 receptor (e.g., CM398).

### 2.2. Modeled Structural Interactions between Sigma Receptors and Ligands Provides a Basis for Antiviral Drug Optimization

Since antiviral activity against SARS-CoV-2 was driven by agonism of the sigma-1 receptor (e.g., SA4503, sigma-1 receptor agonist), but not by antagonism of the sigma-1 receptor (CM304, sigma-1 receptor antagonist), we mapped ligand interactions by molecular docking to identify sites on the sigma-1 receptor that could be used as the basis for optimization of antiviral sigma-1 receptor agonists.

**Figure 5:**
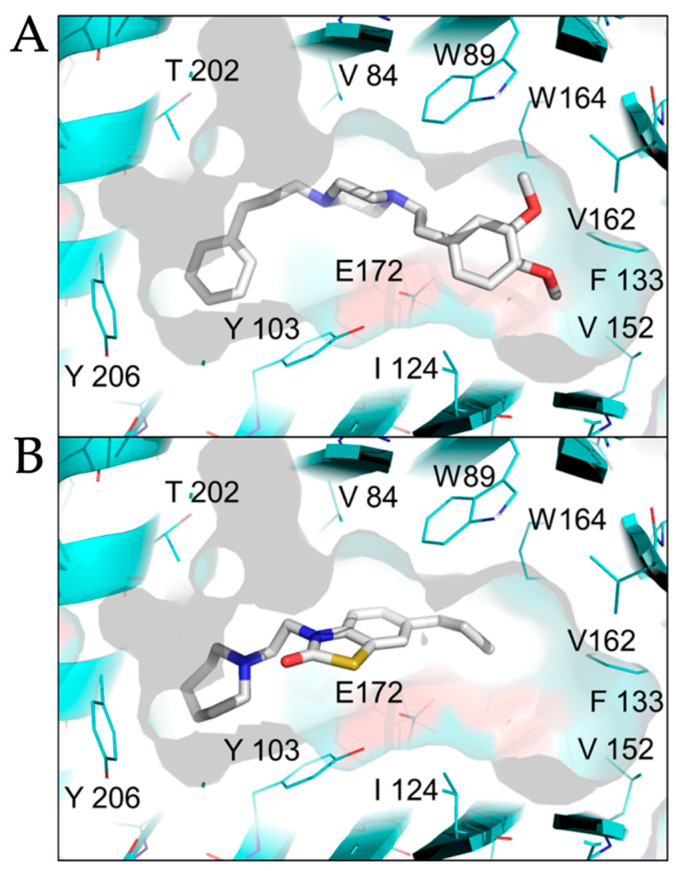
**Comparison of agonist versus antagonist interactions with the sigma-1 receptor by molecular docking.** The crystal structure of the human sigma-1 receptor (PDB 5HK1), shown in cyan, was used as the basis for molecular docking with an selective agonist SA4503 (cutamesine, active against SARS-CoV-2), (**A**, upper panel), and antagonist CM304 (inactive against SARS-CoV-2), (**B**, lower panel). SA4503 and CM304 are shown as sticks, white for carbon, blue for nitrogen, red for oxygen, yellow for sulfur.

The sigma-1 receptor crystal structure shows the C-terminal domain exhibiting a cupin-like β-barrel with a buried, central ligand-binding site [23]. We used the ligand binding site of the sigma-1 receptor (PDB 5HK1) as the basis for molecular docking simulations to compare active antiviral versus inactive sigma-1 receptor ligands. SA4503, an antiviral sigma-1 receptor agonist, was predicted to form contact with multiple residues in the central ligand-binding site of the sigma-1 receptor: V84, W89, Y103, I124, F133, V152, V162, W164, E172, T202 (Figure 5A). In contrast, CM304, a highly specific sigma-1 receptor antagonist, but inactive against SARS-CoV-2, formed contact with a subset of residues in the ligand-binding site of the sigma-1 receptor: V84, W89, Y103, E172, T202 (Figure 5B). These data suggest that sigma-1 receptor binding drugs may be optimized for agonist and antiviral binding activity by forming interactions with specific residues in the sigma-1 receptor ligand-binding site: I124, F133, V152, V162, and W164.

**Figure 6:**
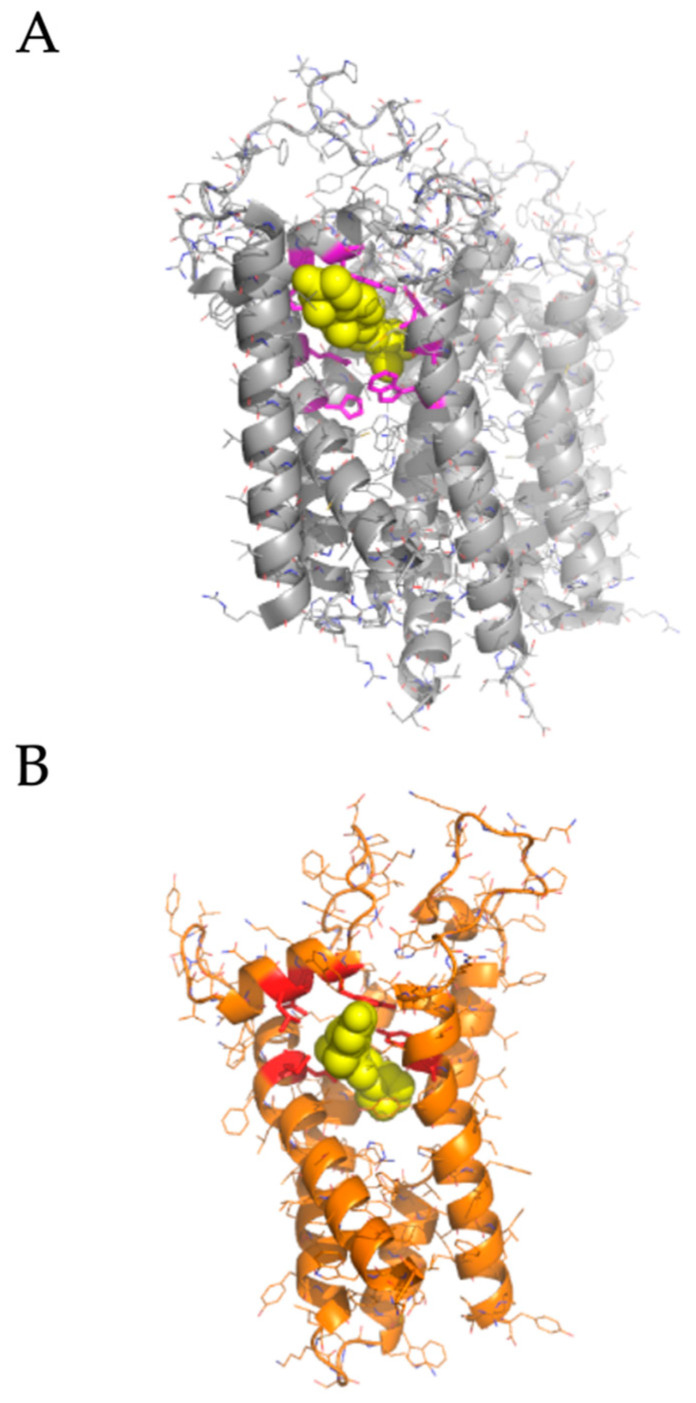
**Homology modeling the human sigma-2 receptor and definition of a putative ligand binding site. (A**), upper panel, the crystal structure of Emopamil-Binding Protein (EBP), PDB 6OHT, shown in gray, was solved complexed to an inhibitor, U18666A, shown as yellow spheres. Ligand binding residues are shown as magenta sticks. (**B**), homology model of the human sigma-2 receptor based on EBP, shown in orange. AZ66, a dual sigma-1 and sigma-2 receptor ligand, is shown as posed by molecular docking using AutoDock Vina as yellow spheres. The putative contact residues on the sigma-2 receptor are shown as red sticks.

**Figure 7:**
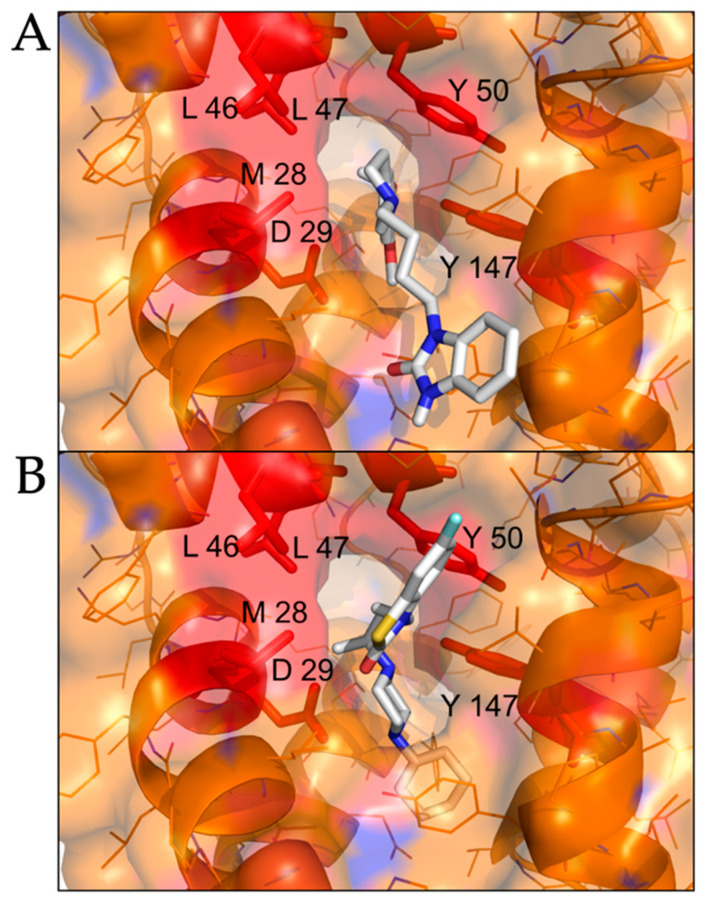
**Molecular docking of sigma-2 receptor ligands that exhibit antiviral activity against SARS-CoV-2.** (**A**), highly selective sigma-2 receptor agonist CM398 is shown as posed by AutoDock Vina to a model of the human sigma-2 receptor. (**B**), dual sigma-1 and sigma-2 receptor ligand AZ66 is shown as posed by molecular docking. Putative interacting residues are shown in red. CM398 and AZ66 are shown as sticks, white for carbon, blue for nitrogen, red for oxygen, yellow for sulfur.

We performed molecular docking simulations of sigma-2 receptor ligands using a homology model of the human sigma-2 receptor. SWISS-MODEL [24] was used to generate atomic coordinates based on the most similar solved structure, 3-β-hydroxysteroid-Δ8,Δ7-isomerase, known as Emopamil-Binding Protein (EBP) [25], PDB 6OHT. EBP, similar to the sigma-2 receptor, is an endoplasmic reticulum membrane protein involved in cholesterol biosynthesis and autophagy. The human sigma-2 receptor, 17.8% identical to EBP, was modeled as a transmembrane protein comprised of α-helices and loop regions that form a putative ligand binding pocket. The structure of EBP was solved complexed to a cholesterol biosynthesis inhibitor U18666A [26], shown as spheres in Figure 5A. Sigma-2 receptor ligands that exhibited antiviral activity against SARS-CoV-2 in vitro were docked against the putative ligand binding site of the modeled sigma-2 receptor structure (Figure 6B). Molecular docking showed that sigma-2 receptor specific ligand CM398, and the sigma-1/sigma-2 receptor ligand AZ66, have the potential to form intermolecular interactions with the ligand-binding site residues (M28, D29, L47, Y50, Y147, shown in red in Figure 7), equivalent to the ligand-binding site residues of EBP (shown in magenta in Figure 6A). These data provide a structural basis for strategies to optimize antiviral activity against SARS-CoV-2 and selectivity for sigma-1/sigma-2 receptor binding.

### 2.3. Synergistic Antiviral Activity by Combining a Sigma Receptor Ligand with Lactoferrin

**Figure 8:**
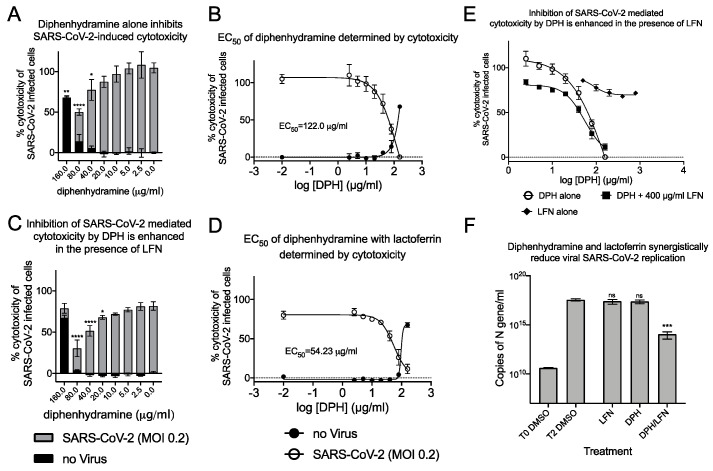
**Combinations of diphenhydramine and lactoferrin exhibit synergy against SARS-CoV-2.** (**A**), Vero E6 cells were treated with diphenhydramine (DPH) at various concentrations without (black bars) or with SARS-CoV-2 at MOI 0.2 (gray bars) and cytotoxicity was measured by LDH release. (**B**), The EC_50_ (white circles) and CC_50_ (black circles) curves were determined by non-linear regression. The EC_50_ of diphenhydramine alone was 122 μg/mL. (**C**), Vero E6 cells were treated with diphenhydramine at various concentrations and lactoferrin (LFN) at 400 μg/mL without (black bars) or with SARS-CoV-2 at MOI 0.2 (gray bars) and cytotoxicity was measured by LDH release. (**D**), The EC_50_ (white circles) and CC_50_ (black circles) curves were determined by non-linear regression. The EC_50_ of diphenhydramine with 400 μg/mL of lactoferrin was 54.25 μg/mL. (**E**), The EC_50_ curves of DPH (white circles), LFN (black diamonds), and DPH+LFN (black squares) are shown on the same graph to compare effect of LFN on DPH EC_50_. (**F**), Measurement of viral genome equivalents by RT-qPCR of the SARS-CoV-2 N-protein gene demonstrate the ability of DPH+LFN to inhibit replication by almost 3-logs. *, *p* ≤ 0.05; **, *p* ≤ 0.01; ***, *p* ≤ 0.001; ****, *p* ≤ 0.0001; ns, not significant.

The antihistamine diphenhydramine, with on-target binding to the Histamine-1 receptor, has known off-target effects at the sigma-1 receptor [27]. Diphenhydramine was recently shown to inhibit SARS-CoV-2 infectivity and the calculated EC_50_ for SARS-CoV-2 by plaque reduction assay was 17.4 μg/mL (59.6 μM). This drug is safe, well-characterized, and widely available and so highly relevant in the search for COVID therapeutics. We investigated the ability of diphenhydramine to inhibit SARS-CoV-2 induced cytotoxicity and found an EC_50_ of 122.0 μg/mL (418 μM; Figure 8A,B), about 7 times higher than that found in the plaque reduction assay, similar to our findings with AZ66. We hypothesized that diphenhydramine could be combined with structurally distinct antiviral agents (binding other receptors, not sigma) to reduce its EC_50_ for antiviral activity against SARS-CoV-2. 

In our investigations into sigma-binding ligands, including diphenhydramine, we sought to reduce the EC_50_ by addition of another safe, and well characterized protein from milk, lactoferrin. The host-iron sequestration protein lactoferrin was reported to exhibit direct antiviral activity against SARS-CoV-2 [28,29], is broadly antimicrobial, and possesses host immunostimulatory properties. We tested combinations of lactoferrin with diphenhydramine to measure effects on reduction of EC_50._ Co-administration of 400 μg/mL of lactoferrin with diphenhydramine further reduced SARS-CoV-2 induced cytotoxicity and decreased the EC_50_ by 55.5% to 54.2 μg/mL (185.7 μM; Figure 8C,D). The antiviral enhancement effects of lactoferrin are more apparent at lower, therapeutically relevant concentrations of diphenhydramine (Figure 8E). Inhibition of viral replication was also investigated by qPCR (Figure 8F). Lactoferrin (400 μg/mL) was able to decrease N-protein RNA copies by 28.0% 48 h after infection, compared to DMSO alone controls while 40 μg/mL diphenhydramine alone resulted in 32.2% reduction. When combined, they inhibited 99.97% of N-protein RNA copies, a 3-log reduction that was highly significant. These data demonstrate that combinations of two over-the-counter compounds, with well characterized safety profiles, have synergistic effects on inhibition of SARS-CoV-2. 

### 2.4. Sigma Ligands Inhibit Infectious Particle Production in Human Lung Cells

**Figure 9:**
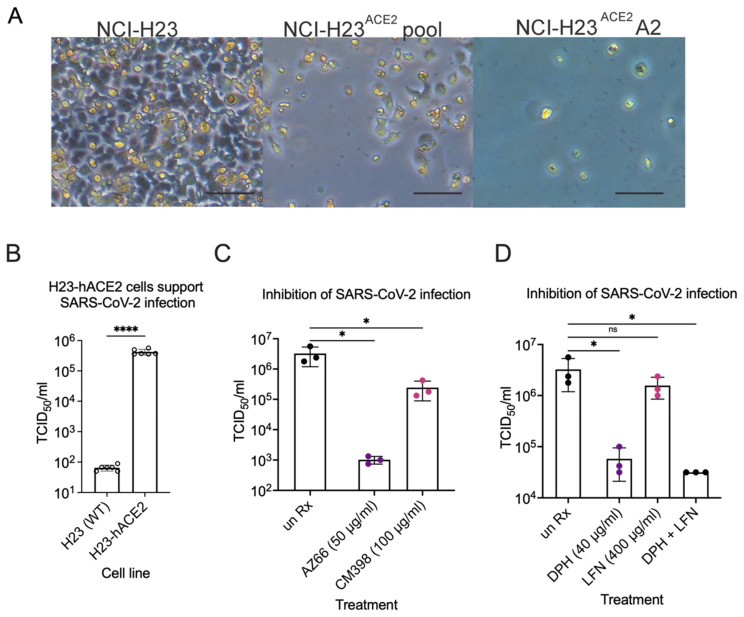
**Inhibition of SARS-CoV-2 infection in human lung epithelial cells.** (**A**) CPE in infected NCI-H23 and NCI-H23ACE2 cells. NCI-H23 (parental untransduced cells), NCI-H23ACE2 pool (lentivirus transformed cells uncloned), and NCI-H23ACE2 (clone A2) were infected with SARS-CoV-2 and CPE was observed 3 dpi. CPE is defined by cell rounding and detachment from the monolayer. The scale bar is equivalent to 100 μm. (**B**), TCID_50_ experiments were performed in biological duplicate three times after infecting cells at an MOI of 0.01 for 72 h. SARS-CoV-2 infection of the human lung epithelial cell line H23 is dependent on heterologous expression of the human ACE2 receptor. (**C**), The mixed affinity sigma-1/sigma-2 receptor ligand AZ66 and the sigma-2 receptor specific ligand CM398, significantly reduce the amount of infectious SARS-CoV-2 particles released from H23-hACE2 cells by ~3-log and 1-log, respectively. Data are from TCID_50_s carried out in technical triplicate. (**D**), Diphenhydramine and diphenhydramine with lactoferrin significantly reduce infectious SARS-CoV-2 particle release from H23-hACE2 cells by ~2-log compared to untreated H23-hACE2 cells. Data are from TCID_50_s carried out in technical triplicate. *, *p* ≤ 0.05; ****, *p* ≤ 0.0001; ns, not significant.

Lastly, inhibition of SARS-CoV-2 infection by compounds shown efficacious in Vero E6 cells was determined in human lung epithelial cells. We generated a new lung cell line susceptible to SARS-CoV-2 infection, H23-ACE2, by lentivirus transduction to introduce the human ACE2 gene. Single clone isolation of the H23-ACE2 transduced cell pool resulted in several healthy clones, including clone A2. Successful ACE2 expression was functionally indicated by increased cytopathic effect upon SARS-CoV-2 infection of an H23-ACE2 cell pool and an isolated cell clone H23-ACE2 clone A2 but not the parental H23 cell line (Figure 9A). ACE2 surface expression was confirmed by flow cytometry as a peak shift to the right on the X-axis towards for H23-ACE2 cell pool and H23-ACE2 clone A2 compared to the untransduced parent H23 cell line and Vero E6 cells. H23-ACE2 clone A2 was used for further experiments. SARS-CoV-2 was used to infect the human lung epithelial cell line H23 at an MOI of 0.01. This cell line is unable to support SARS-CoV-2 infection without heterologous expression of the ACE-2 receptor [30] and our experiments confirmed the essentiality of hACE2 for infection (Figure 9A,B). TCID_50_s were performed to measure infectious particles released during infection in the presence of AZ66, CM398, diphenhydramine, lactoferrin and diphenhydramine+lactoferrin. The mixed affinity sigma-1/sigma-2 receptor ligand AZ66 was able to decrease SARS-CoV-2 concentrations by ~3-log at 48 hpi compared to mock treated-infected H23-ACE2 cells (Figure 9C). Cells were originally infected at an MOI of 0.01 which is equivalent to 1.5 × 10^3^ virus, so the data show AZ66 completely blocks production of infectious virus particles in these experiments. The sigma-2 receptor specific ligand CM398 was able to reduce SARS-CoV-2 concentrations by ~1-log. Diphenhydramine effectively reduced SARS-CoV-2 concentrations by ~2-log while lactoferrin was ineffective (Figure 9D). The combination of diphenhydramine+lactoferrin showed a combined ability to reduce SARS-CoV-2 repication by half that observed for diphenhdydramine alone. The data from the more physiologically relevant human lung cell lines demonstrate the potential for sigma receptor ligands and drugs with off-target effects on sigma receptors to inhibit SARS-CoV-2 replication.

## 3. Discussion and Conclusions

SARS-CoV-2, the causative virus of COVID-19 pandemic, belongs to a family of positive-sense single-stranded RNA (+ssRNA) coronaviruses (CoVs) that also cause illnesses ranging from common colds to severe diseases such as Middle East respiratory syndrome (MERS). There are 7 CoVs known to infect people: 229E, NL63, OC43, HKU1, MERS-CoV, and SARS-CoV that emerged in 2003 [3]. CoV infection is known to activate pathways that facilitate adaptation of ER stress for viral replication [31]. CoVs utilize host cell ER stress responses to modulate protein translation, ER protein folding capacity, ER-associated degradation (ERAD) including autophagy, and apoptotic cell death [32,33]. It has been proposed that modulation of CoV induced ER stress responses may provide the rationale for new approaches to antiviral drug therapy.

Sigma receptors act as modulators of ER stress, functioning as ligand operated membrane bound chaperones at the ER-mitochondrial contact (mitochondrion-associated ER membrane) [34]. Sigma-1 receptor ligands have been shown to exert antiviral activity against CoVs and non-CoVs, including Ebola, HCV, SARS-CoV, SARS-CoV-2, DENV, MERS-CoV, FLUAV (H5N1), HCV, HIV and HSV-1 [10]. Sigma receptors were implicated as targets for antiviral drugs by mapping interactions between human proteins and 26 (of 29) SARS-CoV-2 proteins, and subsequent screening of approved drugs [7]. Two sets of pharmacological agents effectively inhibited SARS-CoV-2 infectivity in Vero E6 cells: inhibitors of mRNA translation and predicted regulators of the sigma-1 and sigma-2 receptors. Non-selective sigma-1 receptor ligands, including the antihistamines clemastine and cloperastine, exhibited activity against SARS-CoV-2 in vitro. PB28 a sigma-1 and sigma-2 receptor ligand was highly efficacious in vitro [7] but was toxic in vivo so the search for effective ligands was continued in this work [35]. 

Mechanisms that drive anti-SARS-CoV-2 activity by sigma receptors are not well characterized. It is not understood if both sigma-1 or sigma-2 receptors are involved in antiviral activity, or if agonism, or antagonism of individual receptors mediate antiviral activity. A significant limitation to addressing the role of sigma receptors in SARS-CoV-2 inhibition (of entry, replication or infectious virus assembly/release) is the paucity of structural information available for the sigma-2 receptor, and absence of well characterized agonists and antagonists. Identification of ligands that exert antiviral activity by specific sigma receptor binding may provide the basis for use of existing drugs (repurposed) and for development of new drugs optimized for activity against CoVs.

Combining a sigma receptor ligand with antiviral drugs that bind distinct targets may provide additive or synergistic antiviral effects and decrease the likelihood of SARS-CoV-2 resistance to a single drug. Drug combinations are recommended for antiviral therapy of hepatitis C (e.g., combination of alpha interferon, simeprevir and ribavirin), and HIV [36]. Combinations of drugs that bind host and/or viral proteins have the potential lessen the severity of COVID-19 by inhibiting virus replication and reducing symptoms. Administration of antiviral drug combinations to SARS-CoV-2 positive patients could determine hospitalization versus home-based care.

Data suggests that specific drugs that bind SARS-CoV-2, or interacting host proteins, also have the potential to prevent COVID-19. For example, hydroxyzine is a first-generation antihistamine that exhibited off-target binding to the SARS-CoV-2 host receptor ACE2 [37] and the sigma-1 receptor. Usage of hydroxyzine (and structurally related antihistamines diphenhydramine and azelastine) was associated with reduced incidence of SARS-CoV-2 positivity in a population of more than 219,000 individuals in California [8]. Hydroxyzine, diphenhydramine and azelastine exhibited direct antiviral activity against SARS-CoV-2 infection of Vero E6 cells in vitro. Since antihistamines act as nasal decongestants and cough suppressants, the on- and off-target binding properties of drugs such as diphenhydramine may have broad utility in prevention and treatment of COVID-19.

In this study, we defined selective sigma receptor ligands (Figure 1) that drive antiviral activity against SARS-CoV-2. The dual specificity sigma-1 and sigma-2 receptor ligand AZ66 exhibited antiviral activity against SARS-CoV-2 induced cytotoxicity of Vero E6 cells (Figure 2A). Since the sigma-1 receptor antagonist CM304 did not inhibit viral cytotoxicity (Figure 1B), and the sigma-1 receptor agonist SA4503 (cutamesine) exhibited inhibitory activity (Figure 2D), these data suggest that sigma-1 receptor agonism drives antiviral activity against SARS-CoV-2. Ligation of the sigma-2 receptor may drive antiviral activity independently, since the highly selective sigma-2 receptor ligand CM398 exhibited direct inhibitory activity against SARS-CoV-2 (Figure 2C). Ligation of both sigma-1 and sigma-2 receptors may elicit higher levels of antiviral activity compared to receptor specific ligands, since AZ66 exhibited the greatest gap between SARS-CoV-2 inhibitory and cellular toxic concentrations (CC_50_/EC_50_ ratio, Table 1). 

We verified the ability of sigma receptor ligands to exhibit antiviral activity by infecting Vero E6 cells at a low MOI and quantifying viral replication by qPCR (Figure 3A). The dual sigma receptor ligand AZ66 exhibited the more significant antiviral effects compared to the selective receptor ligands. AZ66 exhibited antiviral activity against SARS-CoV-2 by plaque assay (Figure 3B). Induction of phospholipidosis by these compounds was measured in human lung epithelial cells (Figure 3C). All compounds induced phospholipidosis to ~50% of the positive control. However, we were unable to identify a correlation between phospholipidosis and inhibition of virus replication and is consistent with findings from other groups [38]. CM304 was a strong inducer of phospholipidosis yet was ineffective at inhibition of SARS-CoV-2. These data indicate that the antiviral activities of sigma ligands AZ66 and CM398 are driven by specific antiviral inhibitory mechanisms outside of phospholipidosis. These data are consistent with microscopic observation of AZ66 (sigma-1 and sigma-2 receptor ligand) and CM398 (sigma-2 receptor ligand) reducing cell rounding and death caused by SARS-CoV-2 infection (Figure 4). 

We mapped potential interactions between ligands and sigma receptors to gain insight in intermolecular interactions that promote antiviral activity against SARS-CoV-2. Identification of specific residues in sigma receptors that bind antiviral drugs may provide the basis for drug development strategies to optimize ligand binding. 

We used a crystal structure of the human sigma-1 receptor (PDB 5HK1) as the basis for molecular docking of sigma-1 receptor ligands. Comparison of the posed orientations of a sigma-1 receptor agonist (SA4503) with an antagonist (CM304) complexed to the sigma-1 receptor revealed that the agonist (with antiviral activity) formed more intermolecular contacts with the receptor compared to the antagonist (without antiviral activity) (Figure 5). These data provide the basis for site directed mutagenesis studies to define key ligand binding residues. These data suggest that drugs optimized for sigma-1 receptor agonist and SARS-CoV-2 antiviral activity may be achieved with analogs that form interactions with specific residues in the sigma-1 receptor ligand-binding site: I124, F133, V152, V162, and W164.

Since crystal structures are not available for the human sigma-2 receptor, we generated an atomic homology model based on the most similar solved structure, 3-β-hydroxysteroid-Δ8,Δ7-isomerase, known as Emopamil-Binding Protein (EBP). EBP, a transmembrane protein comprised of α-helices and loop regions that form a ligand binding site, was solved complexed to an inhibitor (Figure 6A) [25]. We used molecular docking to simulate ligand binding of sigma-2 receptor specific ligand CM398, and sigma-1/sigma-2 receptor ligand AZ66, predicted to form intermolecular interactions with ligand-binding site residues M28, D29, L47, Y50, Y147, Figure 5B. In addition to the anti-viral effects of AZ66, binding of the sigma receptors reduces nociception [17]. The analgesic effect of AZ66 could provide novel treatment of SARS-CoV-2 related pain while inhibiting viral replication. These data provide the basis for mutagenesis and structure-activity-relationship studies to optimize sigma-2 receptor binding and antiviral activity against SARS-CoV-2.

Specific antihistamines exhibit off-target sigma receptor binding activity, and also exhibit antiviral activity against SARS-CoV-2, including clemastine, cloperastine, astemizole, hydroxyzine, azelastine and diphenhydramine. Since diphenhydramine is the most commonly used antihistamine exhibiting antiviral activity, we asked if antiviral activity could be improved by combining a sigma receptor ligand with lactoferrin, an antiviral agent that binds distinct targets [28,29]. We found that co-administration of 400 μg/mL of lactoferrin with diphenhydramine reduced SARS-CoV-2 induced cytotoxicity and decreased the EC50 (Figure 8C,D). The antiviral enhancement effects of lactoferrin were more apparent at lower, therapeutically relevant concentrations of diphenhydramine (Figure 8E). Combining lactoferrin with diphenhydramine resulted in synergistic effects on antiviral activity against SARS-CoV-2 (Figure 8F). Compounds we found effective in Vero E6 were validated in their ability to reduce infectious SARS-CoV-2 production following infection of human lung epithelial cells (Figure 9C,D). These data suggest that sigma receptor ligands or formulated combinations of over-the-counter products have the potential to inhibit virus infection and/or decrease recovery time from COVID. Lastly, concentrations that inhibited SARS-CoV-2 production were decoupled from phospholipidosis in human lung epithelial cells, suggesting a specific mechanism at the sigma receptors/virus interface. The candidates investigated in this work target sigma receptors that result in selectivity indices higher than remdesivir, a top candidate in large-scale in silico screens that showed efficacy in vitro and in vivo [6]. 

## 4. Materials and Methods

### 4.1. Sigma Ligands and Other Drugs Used in this Study 

AZ66, CM304, and CM398 were synthesized and characterized in the McCurdy lab at the University of Florida as previously reported [17,22,39] with purities > 95% each (Supplementary Materials). SA 4503 (cutamesine) was obtained from MilliporeSigma (St. Louis, MO, USA) at >98% pure according to the manufacturer and diluted in PBS to 2 mg/mL and frozen at −80 °C in aliquots to eliminate freeze thaw cycles. Lactoferrin from human milk was obtained from MilliporeSigma at >85% purity according to the manufacturer and diphenhydramine HCl was purchased from Spectrum Pharmaceuticals at ≥98% purity according to the manufacturer.

### 4.2. Virus Culture Methods

The SARS-CoV-2 strain used in this study was UF-1. It has been described previously [1] and was isolated from a COVID19 patient at UF Health Shands Hospital via nasal swab. Virus experiments were carried out under a University of Florida Institutional Biosafety Committee-approved protocol in a Biosafety Level 3 laboratory at the Emerging Pathogens Institute. The accession number of the previously sequenced strain can be found under the following GenBank accession number: MT295464.1. Vero E6 cells purchased from ATCC were used to propagate virus using standard methods. Vero E6 cells were grown in DMEM + 2%FBS+ PenStrep. SAEC, H23 and H23-hACE2 cells were grown in RPMI + 10% FBS + PenStrep with 4 μg/mL of blasticidin to maintain ACE2 expression if needed. Cell were grown at 37 °C and 5% CO_2_ in a humidified incubator. An EVOS XL Core microscope was used to visualize cells in the BSL3.

### 4.3. Quantitation of Virus Replication by qPCR

SARS-CoV-2 was used to infect Vero E6 monolayers at an MOI of 0.01 in the presence of each treatment in biological and technical triplicate. At 2 days post-infection (dpi), the monolayers were scraped and harvested into viral lysis buffer (buffer AVL) from the QIAamp Viral RNA Kit (QIAGEN). The AVL buffer is a CDC approved method of viral inactivation. Samples were frozen at −80 °C and removed from the BSL3. RNA was purified according to the manufacturer’s recommendations. Reverse transcription and cDNA synthesis was accomplished using the iTaq Universal SYBR Green One-Step Kit (BioRad) and primers targeting the nucleocapsid (N) gene of SARS-CoV-2 (NproteinF- GCCTCTTCTCGTTCCTCATCAC, NproteinR-AGCAGCATCACCGCCATTG). qPCR was carried out on a BioRad CFX96. N protein copy levels were calculated using CT values from a standard curve generated using a control plasmid containing the N protein gene and are presented as genome equivalents (GE) (Integrated DNA Technologies). 

### 4.4. Sigma Ligand Cytotoxicity Reduction Assays

Vero E6 cells were seeded into 96-well CellBind treated plates (Corning) and allowed to attach overnight. Drugs were pre-aliquoted in DMEM + 2%FBS. Cells and drug dilutions were transported into the BSL3 laboratory where titered SARS-CoV-2 aliquots were diluted to produce a target MOI of 0.2 PFU/cell in solution at the final indicated drug concentrations. Triplicate monolayers were infected by replacing growth media with 100 μL of the drug/virus suspensions. At 72 h post infection, supernatants were harvested, and lactate dehydrogenase (LDH) release was assayed using the Cytox 96™ Non-Radioactive Cytotoxicity Assay (Promega, Madison, WI, USA). Assays were performed as recommended by the manufacturer to generate a formazan dye. The optical density at 450 nm was measured using a MultiSkan FC plate reader (Thermo Fisher Scientific, Waltham, MA, USA). Controls included total LDH release as measured by lysis of all cells, spontaneous release from uninfected cells, and media alone. The toxicity of sigma ligands alone were also determined in parallel to discriminate the amount of SARS-CoV-2-induced cytotoxicity occurring in the presence of a given treatment. After spontaneous and background subtraction, OD_450_ values were transformed to a percent of SARS-CoV-2 infected cells (100%) in the absence of any drug treatment to obtain percent of SARS-CoV-2-induced cytotoxicity. These experiments were carried out twice. 

### 4.5. Plaque Reduction Assay

Vero E6 cells were plated in 24-well plates with triplicate replicates on different plates. Virus master mix was used to dilute down to 20-200 PFU/mL and aliquoted in separate tubes with drugs at the final indicated drug concentrations. The drug virus mixtures were immediately used to infect Vero E6 monolayers for 1 h with rocking every 10 min. Monolayers were then overlaid with MEM in 1.5% low-melt agarose containing drugs at the final concentrations indicated. Plaques were counted at 72 hours post infection and used to calculate the apparent reduction in viral concentration compared to the starting volume. Data presented is representative of two independent experiments. 

### 4.6. Generation of ACE-2 Lentivirus Particles 

The lentivirus containing ACE2 were generated by co-transfecting psPAX2, pMD2.G, and an ACE expression vector that also contained a blasticidin selection gene EX-U1285-Lv197 (GeneCopoeia). The plasmids were transfected into HEK293T cells using X-tremeGENE 9 (Roche Cat# XTG9-RO) as per the manufacturer’s instructions. Media was replaced with DMEM containing 2% (*w/v*) bovine serum albumin (BSA) 18 h post transfection and then lentiviruses were collected after 24 and 48 h [40]. 

### 4.7. ACE2 Transduction of NCI-H23 Cells and Monoclonal Cell Selection

NCI-H23 (aka H23) cells were obtained from ATCC (CRL-5800) and ACE2 lentiviruses were filtered through a 0.45 μm filter and used to transduce H23 cells using reverse transduction. Briefly, filtered virus particles are added to the H23 cell suspension with RPMI 1640 (Gibco Cat# 1185093) media supplemented with 10% FBS and 8 μg/mL polybrene (Sigma Cat# TR-1003-G). 72 h post transduction, media was changed to RPMI 1640 supplemented with 10% FBS and 4 μg/mL blasticidin S hydrochloride- (Gibco Cat# R21001). Cells were expanded in increasingly larger cell culture plates and ACE2 expression was confirmed by infecting with SARS-CoV-2 (strain Canada/ON/VIDO-01/2020) and flow cytometry. Single clone isolation from the H23-ACE2 cell pool was carried out by the array dilution method in 96-well plates. Single clones were collected 2-3 weeks after seeding and expanded in increasingly larger cell culture plates. After successful isolation, cells were maintained with complete media containing 2 μg/mL blasticidin.

### 4.8. Analysis of Cell Surface ACE2 by Flow Cytometry

Healthy cells were detached from the monolayer using 0.5 mM EDTA in PBS and centrifuged at 1500 rpm for 3 min. The cell pellet was stained for 1 h at 4 °C with primary ACE2 antibody (R&D systems Cat #AF933, used at a concentration of 0.25 μg/10^6^ cells). The cells were then washed twice with flow wash buffer (2% FBS in PBS) and stained with secondary Goat IgG APC conjugated antibody (R&D systems Cat# F0108, at recommended volume of 10 μL/10^6^ cells), 1000x live-dead viability stain (Invitrogen Cat #L34958) and fixed with 2% PFA (diluted in flow wash buffer). The cells were analyzed using a Beckman CytoFLEX Flow Cytometer and the CytoExpert software.

### 4.9. TCID_50_ Assays in H23 Cells

H23 or H23-hACE2 cells were seeded at 1.5 × 10^5^ cells in Corning CellBIND 24-well plates and allowed to attach overnight. The next day, SARS-CoV-2 was used to infect the cells at an MOI of 0.01 in the presence of mock treatment (PBS), AZ66 (50 μg/mL), CM398 (100 μg/mL), diphenhydramine (40 μg/mL), lactoferrin from human milk (400 μg/mL), or a combination of diphenhydramine (40 μg/mL) and lactoferrin (400 μg/mL). The TCID_50_s were performed by diluting 48 h supernatant from the H23 infections across 8 columns of Vero E6 cells in three independent experiments. Five days later the TCID plates were observed by microscopy for CPE. TCID_50_/mL in the original H23 infection culture supernatant were calculated by the method of Spearman-Kärber. The TCID experiments were carried out in technical triplicate as described above with individual TCID_50_/mL values and their average and standard deviation shown. 

### 4.10. Inhibitory Concentration and Effective Concentration Calculations

Regardless of whether the assay was cytotoxicity or plaque reduction, CC_50_ values and EC_50_ values were calculated using the GraphPad Prism 9 software nonlinear regression module. 

### 4.11. Molecular Docking of Sigma Receptor Ligands 

Sigma receptor ligands were docked individually using AutoDock Vina ^42^ into the ligand binding site of the sigma-1 receptor (PDB 5HK1). The SMILES string of each compound was translated into 3 dimensional coordinates using the NCI/CADD translator (http://cactus.nci.nih.gov/translate/, accessed on 1 October 2021). AutoDock Tools [41] assigned hydrogen atoms and calculated atom charges for AutoDock Vina. Atomic coordinates for ligand PD144418 and solvent molecules were extracted from the sigma 1 receptor structure and each compound was docked to the ligand binding site using AutoDock Vina. The top 9 scoring orientations were evaluated by visual inspection with the highest scoring poses reported. PyMol (https://pymol.org/2/, accessed on 1 October 2021) was used to measure interatomic distances and identify sigma-1 receptor residues implicated in ligand binding.

An atomic model of the human sigma-2 receptor was generated using SWISS-MODEL based on the most similar solved structure, 3-β-hydroxysteroid-Δ8,Δ7-isomerase, also known as Emopamil-Binding Protein (EBP), PDB 6OHT. EBP was solved complexed to an inhibitor, U18666A, and provided the basis for a putative sigma-2 receptor binding site. AutoDock Vina was used for molecular docking simulations of sigma-2 receptor ligands to the modeled human sigma-2 receptor. Figures generated with PyMol.

### 4.12. Phospholipidosis Assay

Phospholipidosis assay was performed with the acCELLerate GmbH (Hamburg, Germany) InstaCELL^®^ Phospholipidosis assay kit per the manufacturer’s protocol using H23 cells. The cells were seeded in a 96 well plate at a seeding density of 1.5 × 10^4^ cells per well and allowed to grow in an incubator at 37 °C, 5% CO_2_. After 24 h, cells were treated with 50 μg/mL of AZ66 and 100 for each of CM304, CM398 or SA4503, along with positive (sertraline, 5 µM) and vehicle (DMSO, 0.5%) controls, and incubated for 48 h. At the end of incubation, cells were washed with PBS buffer and stained with PLD staining solution (LysoID/Hoechst phospholipids staining solution) for 30 min. Thereafter the cells were washed with PBS buffer, and fluorescence was measured using a microplate reader (SpectraMax iD3, Molecular Devices LLC) at 540 nm excitation/680 nm emission and normalized against fluorescence at 340 nm excitation/ 480 nm emission.

## 5. Patents

D.A. Ostrov and M.H. Norris have filed provisional patents for diphenhydramine (#63/070,124) and diphenhydramine plus lactoferrin (#63/126,082) treatment of COVID19. D.A. Ostrov, C. R. McCurdy, and M.H. Norris have filed a provisional patent for use of sigma receptor ligands to treat COVID19 (#63/145,807).

## Figures and Tables

**Table 1:** Cytotoxicity and plaque reduction values of sigma receptor ligands.

	Cytotoxicity					Plaque Reduction	
	CC_50_		EC_50_			EC_50_	
	µg/mL	R^2^		µg/mL	R^2^	µg/mL	R2
AZ66	127.6 (109.8–173.7)	0.9957		45.14 (36.91–55.21)	0.8874	6.47 (1.27–32.86)	0.7369
CM398	110.9 (ND)	0.9618		51.31 (43.78–61.13)	0.9629		
SA4503	198.7 (ND)	0.9401		110.5 (39.2–311.3)	0.8789		

## Data Availability

The data presented in this study are available in the figures, tables, and supplementary files associated with this manuscript.

## Supplementary Materials

The following are available online at https://www.mdpi.com/article/10.3390/pathogens10111514/s1, Figure S1: Proof of purity for compounds AZ66, CM304 and CM398.
