# Supplementary material for: Highly Specific Sigma Receptor Ligands Exhibit Anti-Viral Properties in SARS-CoV-2 Infected Cells
Source: Pathogens. 2021 Nov 20;10(11):1514. doi: 10.3390/pathogens10111514 (PMC8620039; doi:10.3390/pathogens10111514)

LCMS traces of tested compounds with peak purity

LCMS method:

Solvent A: ACN (0.1 % FA); Solvent B: Water (0.1% FA);

Time (min)	Flow (ml/min)	%A	%B
Initial	0.300	10.0	90.0
0.50	0.300	10.0	90.0
3.50	0.300	100.0	0.0
4.00	0.300	100.0	0.0
4.50	0.300	10.0	90.0
6.00	0.300	10.0	90.0

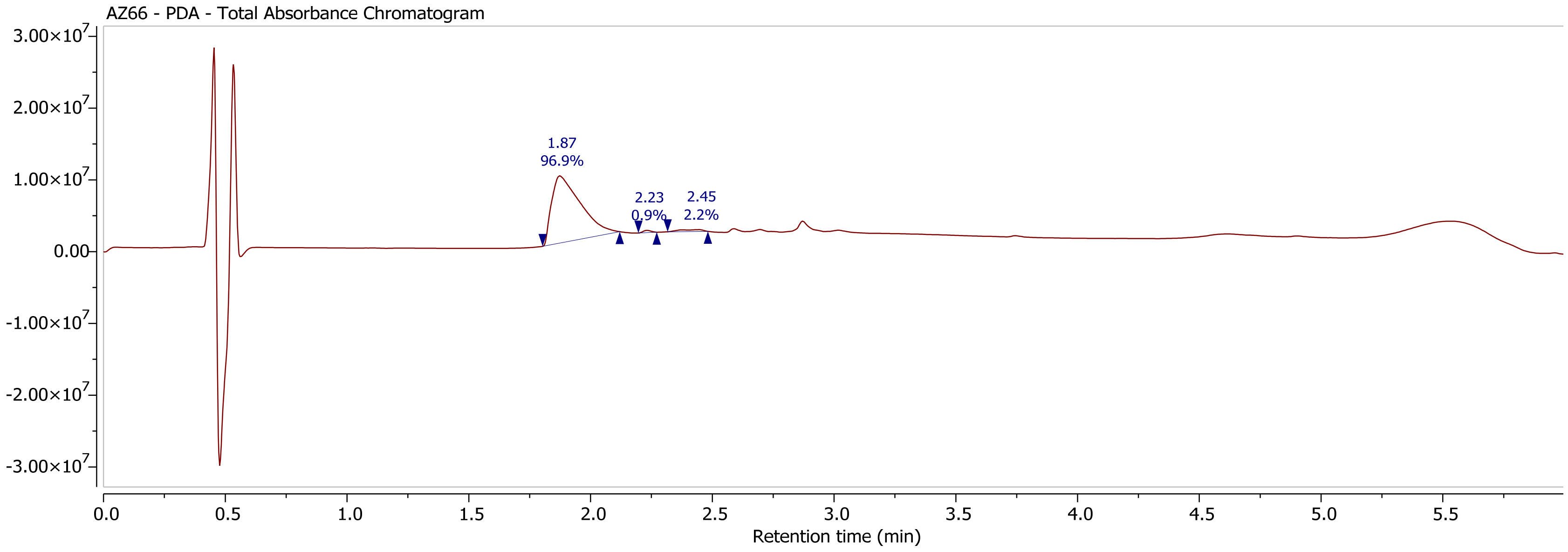
AZ66 - PDA - Total Absorbance Chromatogram
3.00×10^7
2.00×10^7
1.00×10^7
0.00
-1.00×10^7
-2.00×10^7
-3.00×10^7
1.87
96.9%
2.23
0.9%
2.45
2.2%
0.0
0.5
1.0
1.5
2.0
2.5
3.0
3.5
4.0
4.5
5.0
5.5
Retention time (min)

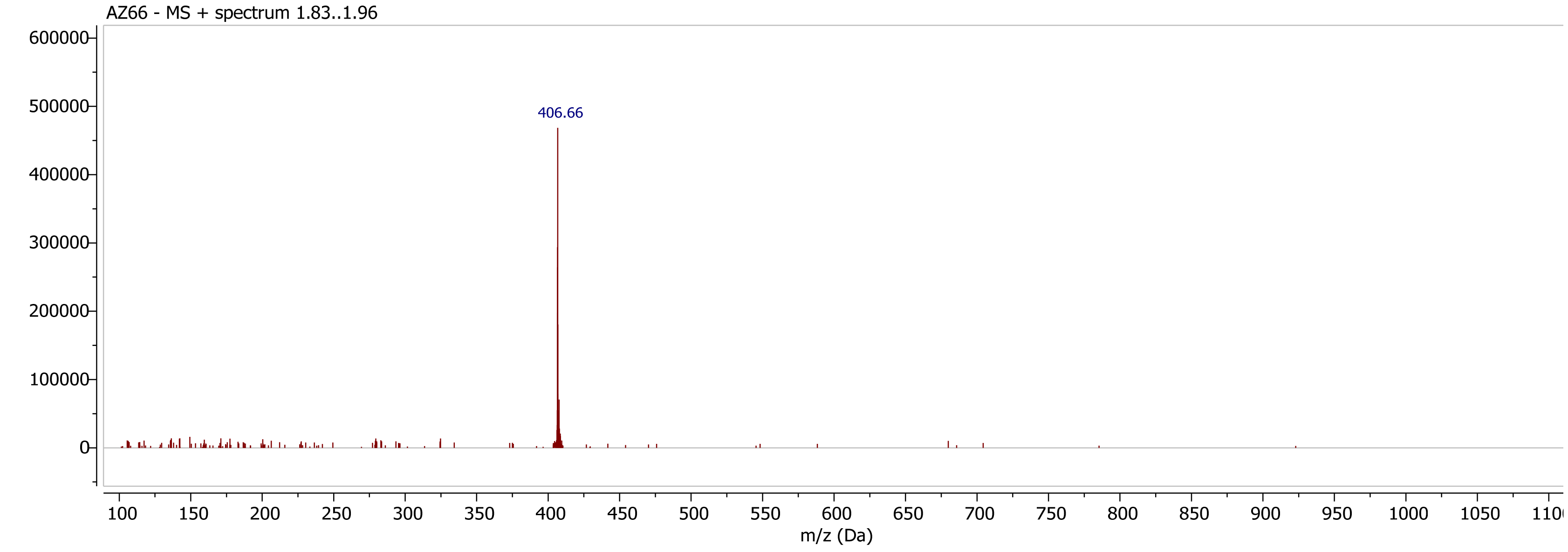
AZ66 - MS + spectrum 1.83..1.96
600000
500000
400000
300000
200000
100000
0
406.66
100
150
200
250
300
350
400
450
500
550
600
650
700
750
800
850
900
950
1000
1050
m/z (Da)

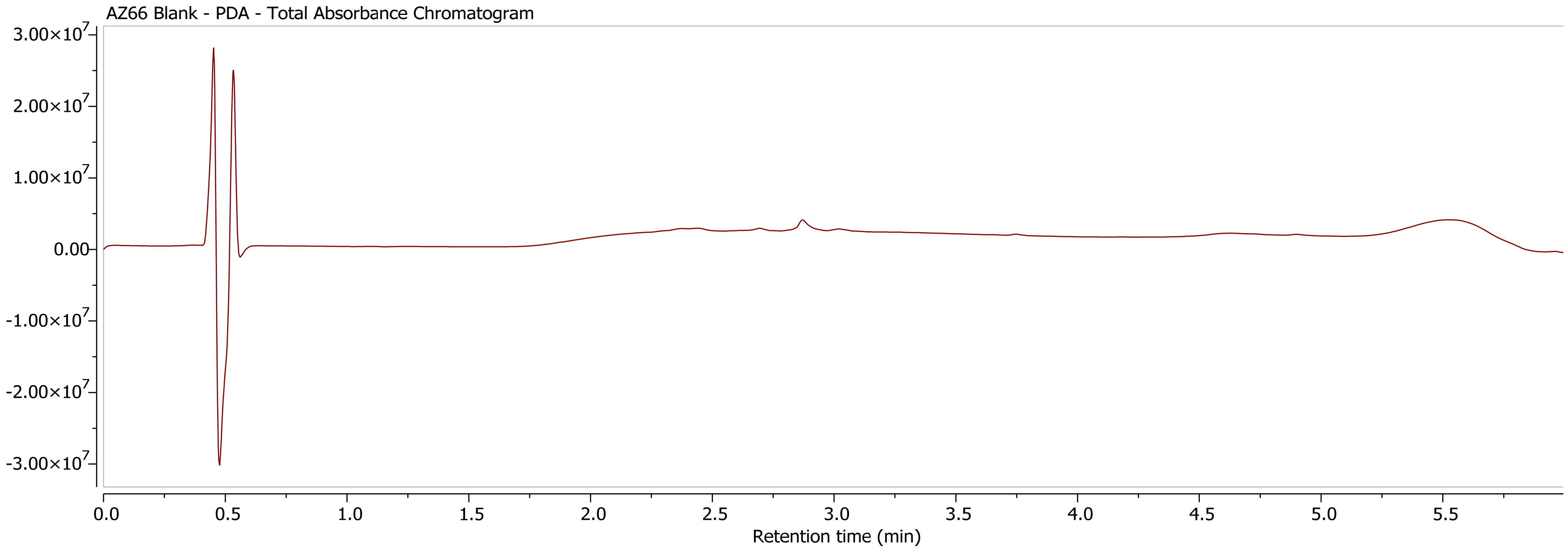
AZ66 Blank - PDA - Total Absorbance Chromatogram
3.00×10^7
2.00×10^7
1.00×10^7
0.00
-1.00×10^7
-2.00×10^7
-3.00×10^7
0.0 0.5 1.0 1.5 2.0 2.5 3.0 3.5 4.0 4.5 5.0 5.5
Retention time (min)

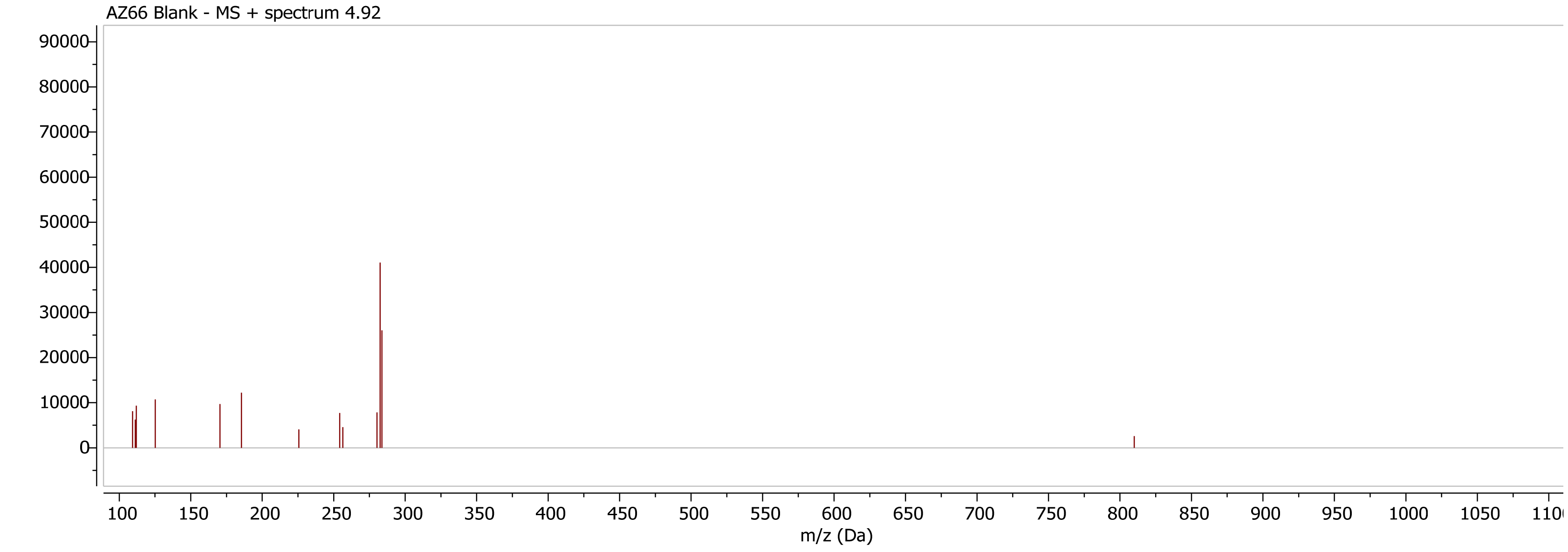
AZ66 Blank - MS + spectrum 4.92
90000
80000
70000
60000
50000
40000
30000
20000
10000
0
100 150 200 250 300 350 400 450 500 550 600 650 700 750 800 850 900 950 1000 1050
m/z (Da)

CM304 - PDA - Total Absorbance Chromatogram

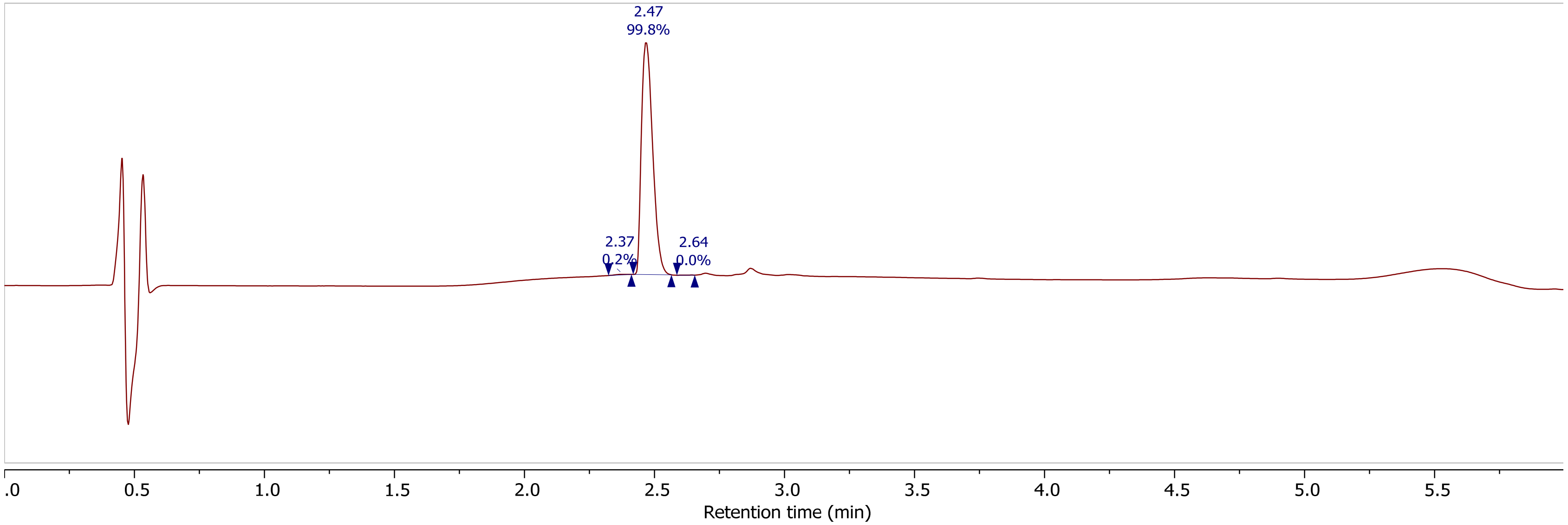

CM304 - MS + spectrum 2.45..2.49

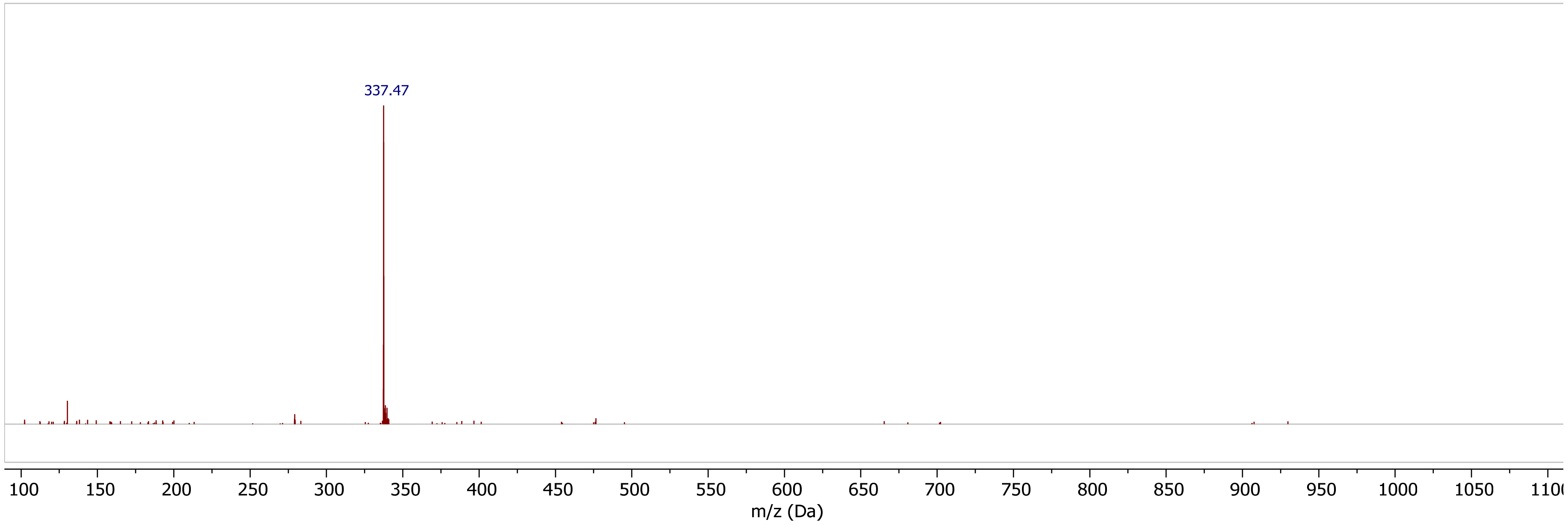

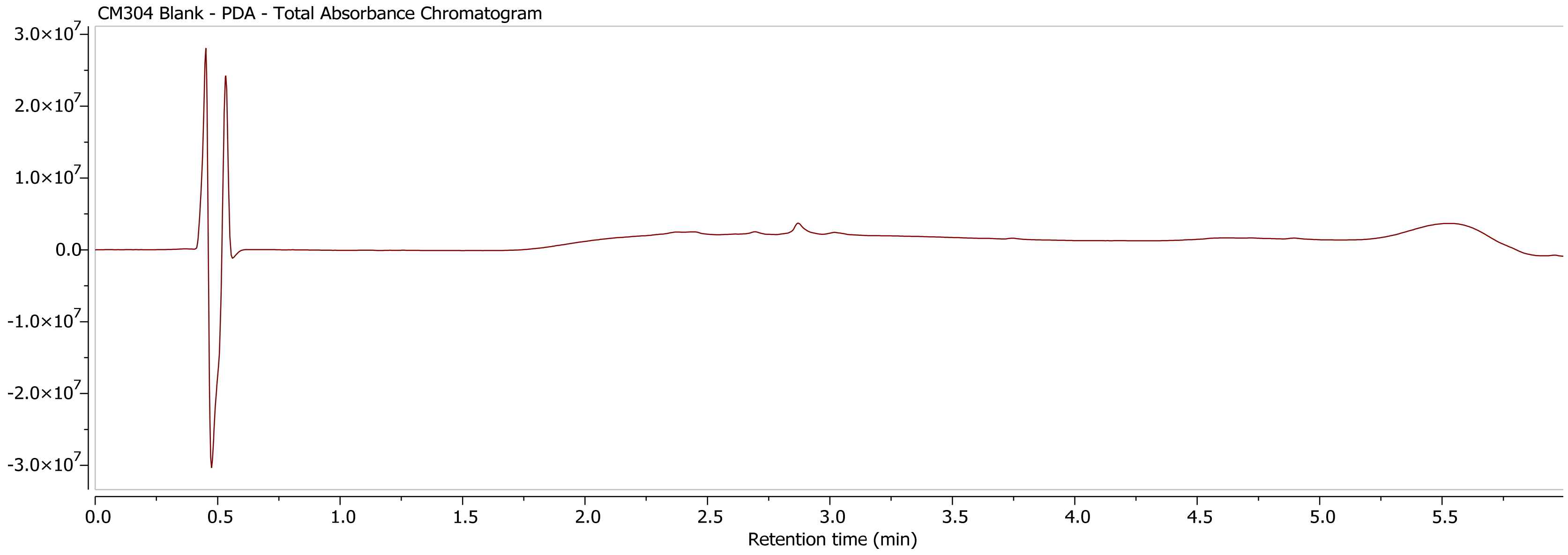
CM304 Blank - PDA - Total Absorbance Chromatogram
3.0×10^7
2.0×10^7
1.0×10^7
0.0
-1.0×10^7
-2.0×10^7
-3.0×10^7
0.0 0.5 1.0 1.5 2.0 2.5 3.0 3.5 4.0 4.5 5.0 5.5
Retention time (min)

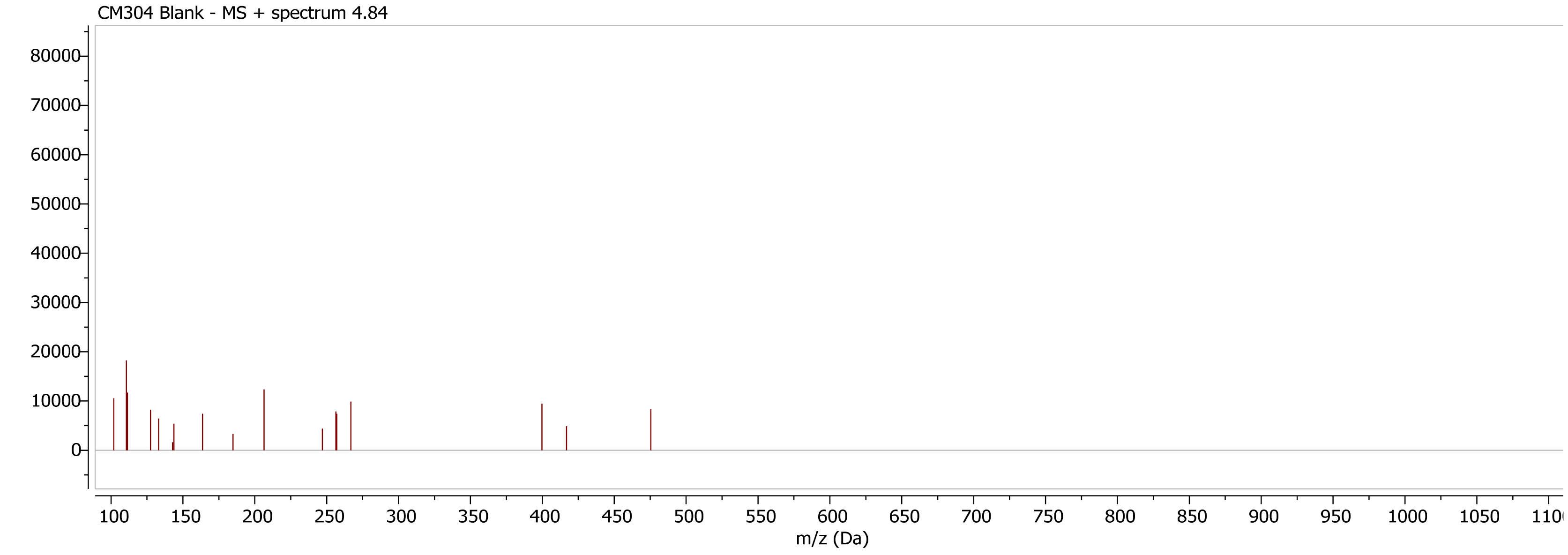
CM304 Blank - MS + spectrum 4.84
80000
70000
60000
50000
40000
30000
20000
10000
0
100 150 200 250 300 350 400 450 500 550 600 650 700 750 800 850 900 950 1000 1050
m/z (Da)

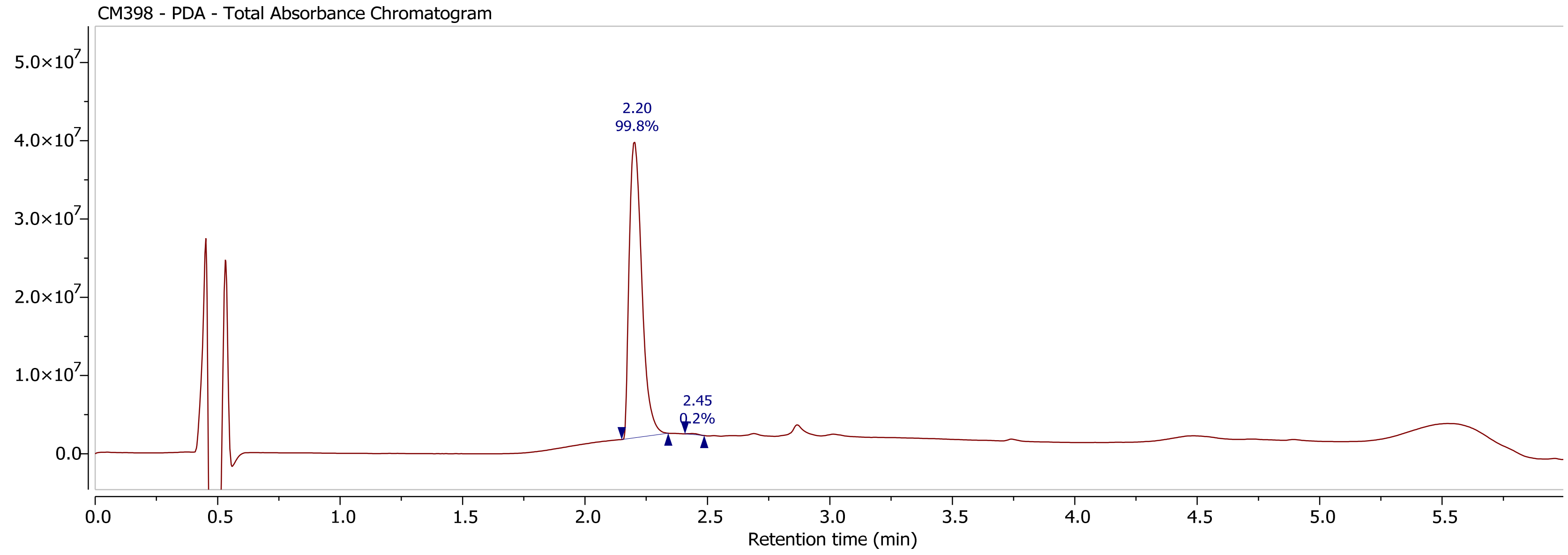
CM398 - PDA - Total Absorbance Chromatogram
2.20
99.8%
2.45
0.2%
Retention time (min)

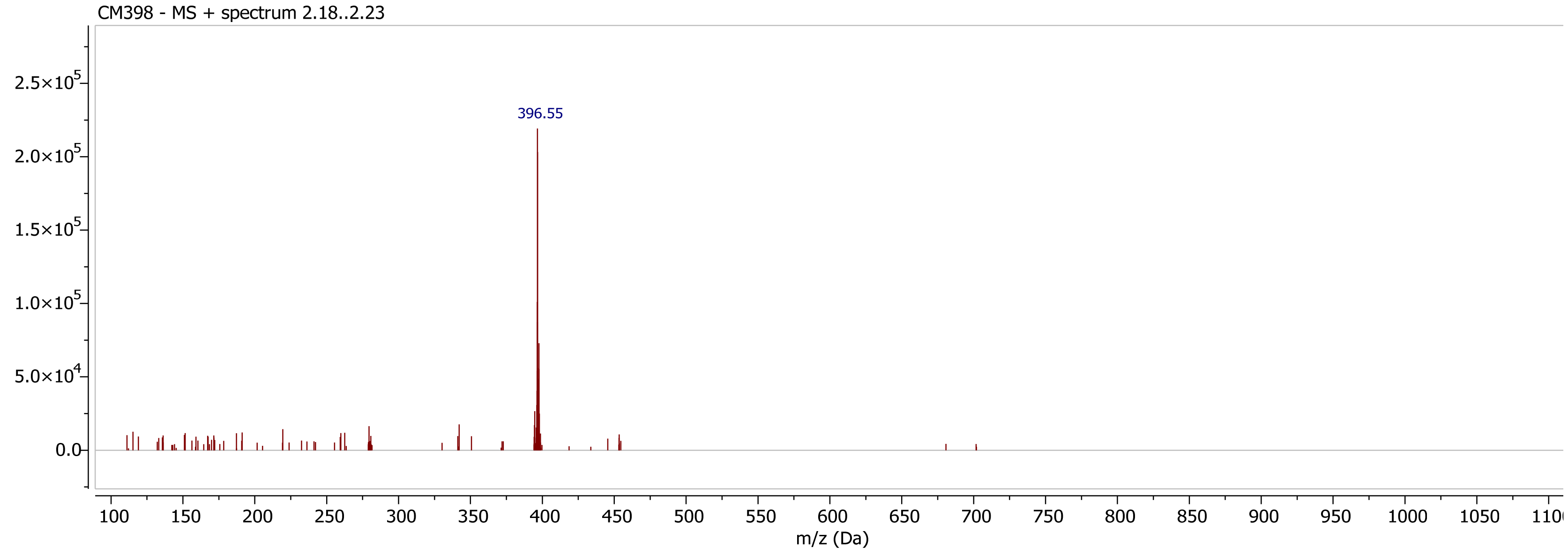
CM398 - MS + spectrum 2.18..2.23
396.55
m/z (Da)

CM398 Blank - PDA - Total Absorbance Chromatogram

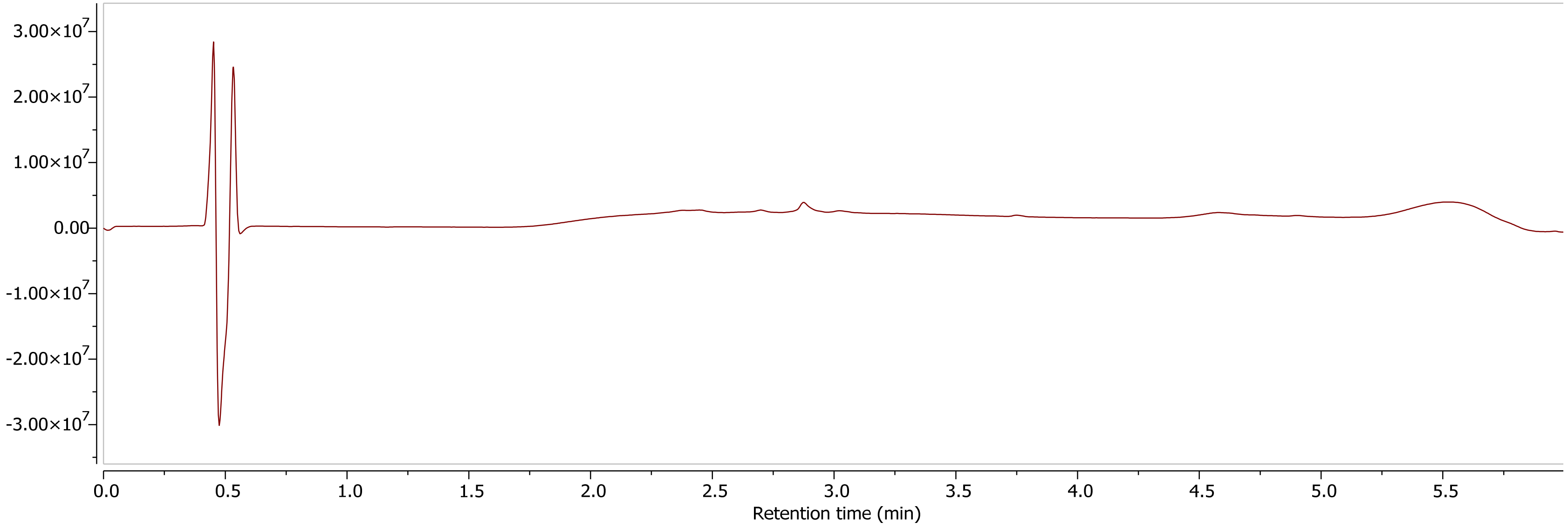

CM398 Blank - MS + spectrum 5.53

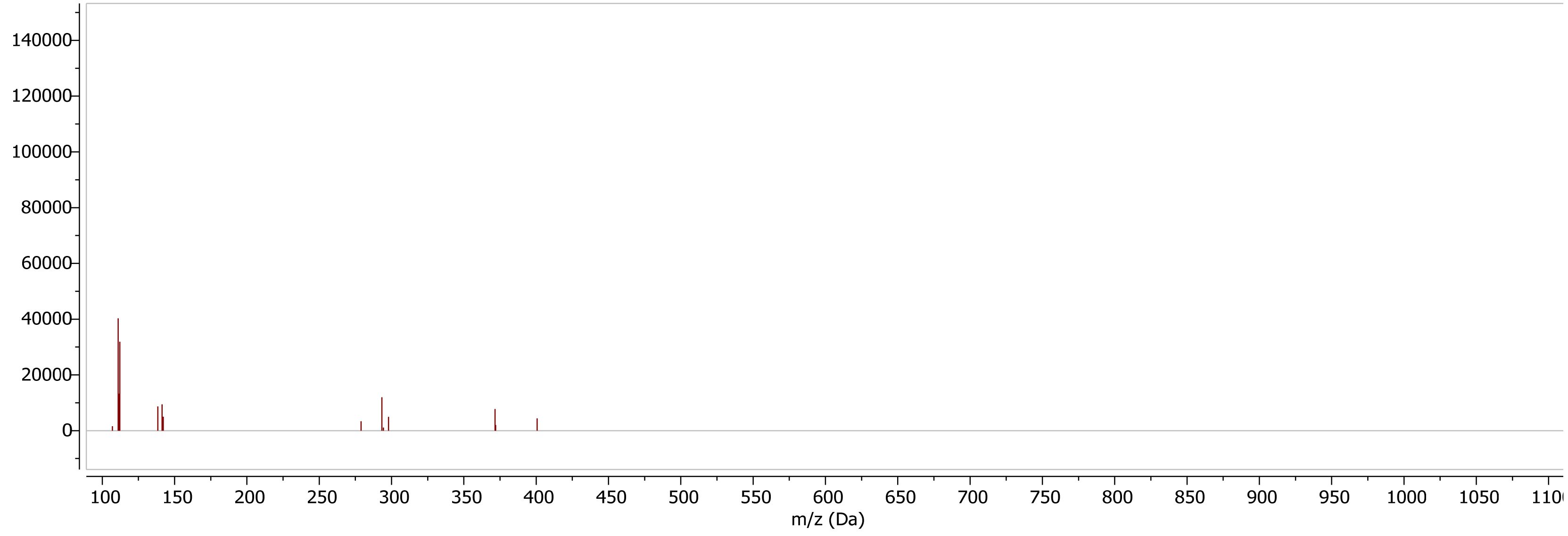

Supplement: Supplementary file 1 [file pathogens-10-01514-s001.zip › pathogens-1442390-supplementary.pdf]
